# Factors affecting the fruit and vegetable intake in Nepal and its association with history of self-reported major cardiovascular events

**DOI:** 10.1186/s12872-020-01710-y

**Published:** 2020-09-24

**Authors:** Sajama Nepali, Anupa Rijal, Michael Hecht Olsen, Craig S. McLachlan, Per Kallestrup, Dinesh Neupane

**Affiliations:** 1 Nepal Development Society, Chitwan, Nepal; 2 Department of Internal Medicine, Holbæk Hospital, Holbæk, Denmark; 3 School of Health, Torrens University, Sydney, NSW Australia; 4 Research Unit for Global Health, Department of Public Health, Aarhus University, Aarhus, Denmark; 5 Welch Center for Prevention, Epidemiology, and Clinical Research, Department of Epidemiology, Johns Hopkins University, Baltimore, MD USA

**Keywords:** Association, Fruit and vegetable, Nepal, Myocardial infarction, Self-reported, Stroke

## Abstract

**Background:**

The World Health Organization recommends consumption of a minimum of 400 g of fruits and vegetables per day for prevention of cardiovascular disease. Low fruit and vegetable intake is associated with an increased risk of stroke by 11% and ischemic heart disease by 31%. The present study aims to explore factors affecting the fruit and vegetable intake in Nepal and its association with history of self-reported major cardiovascular events (myocardial infarction and stroke).

**Method:**

Data for this cross-sectional study were collected as part of the study “Community Based Management of Hypertension in Nepal” initiated in the Lekhnath Municipality in 2013. Demographic and nutrition information were collected using the WHO STEPwise approach to a surveillance tool. Descriptive statistics identified the frequency and percentage of fruit and vegetable intake. A Chi-square test examined the association between fruit and vegetable intake and history of self-reported cardiovascular events, socio-demographic and cardiovascular risk factors. Binary logistic regression analysis identified odds ratio with 95% confidence intervals between fruit and vegetable intake and history of self-reported cardiovascular events.

**Results:**

The mean and median intake of fruits and vegetables were 3.3 ± 0.79 and 3 servings respectively. Of the 2815 respondents, 2% (59) reported having a history of major cardiovascular events. The adjusted odds of having a history of major cardiovascular events was 2.22 (95%CI, 1.06–4.66) for those who consumed < 3 servings compared to those who consumed ≥3 servings of fruits and vegetables a day.

**Conclusion:**

The respondents who consumed < 3 servings of fruits and vegetables a day had higher odds of a history of major cardiovascular events in comparison to those who consumed ≥3 servings. This finding may carry a policy recommendation for those settings where the current recommendation of having ≥5 servings of fruits and vegetables a day is not possible. Our findings also suggest that surviving a major cardiovascular event was not enough in itself to modify nutritional intake. As many Nepali consumes low amount of fruits and vegetables, appropriate measures should be taken to increase this consumption to prevent cardiovascular morbidity and mortality.

## Background

Fruits and vegetables are an essential part of a healthy and balanced diet [1, 2]. Vitamins, minerals, fibers and phytochemicals in fruits and vegetables provide health advantages [1] and contribute to reduce risk factors for cardiovascular diseases [3]. The World Health Organization (WHO) recommends consumption of a minimum of 400 g, corresponding to five servings of fruits and vegetables excluding potatoes and other tuberous root vegetables a day, to prevent cardiovascular diseases [4]. Low intake of fruits and vegetables is estimated to be responsible for 11% of strokes and 31% of ischemic heart disease cases [5]. WHO estimates that 2.7 million lives could be saved each year if fruits and vegetables are consumed adequately [5].

In line with WHO recommendations, studies have reported an inverse association between fruit and vegetable intake and cardiovascular diseases with an intake of 3 to 5 servings and more than 5 daily servings of fruits and vegetables significantly lowered the risk of cardiovascular diseases compared to consuming less than 3 servings a day [6]. Similarly, another study among female health professionals reported that a high intake of fruits and vegetables had a protective effect against cardiovascular diseases [7].

However, very few studies have documented fruit and vegetable intake in low-income countries like Nepal [8]. A nationwide study in Nepal conducted in 2013 reported that almost the entire study population (99%) had an insufficient fruit and vegetable intake [9]. To the best of our knowledge, no studies have explored the association between fruit and vegetable intake and major cardiovascular events in Nepal. The aim of the present study was to explore the factors affecting fruit and vegetable intake in Nepal and its association with cardiovascular diseases assessed as self-reported history of major cardiovascular events (myocardial infarction or stroke).

## Methods

Data of the current study were retrieved from the “Community Based Management of Hypertension in Nepal (COBIN)” study initiated in the former Lekhnath Municipality in 2013 [10], now merged into Pokhara Municipality after federalism in 2015. The detailed protocol and methodology of the project have been published elsewhere [10].

### Study area

The study area was a semi-urban area of Pokhara Municipality (formerly known as Lekhnath Municipality) in the Kaski district of the Gandaki Province of western Nepal. Nepal has a diverse topography and has favorable climate for growing varieties of fruits and vegetables. A number of fruits and vegetables such as apple, peach, pear, plum, walnut, orange, mango, lichi, banana, pineapple, papaya, green leafy vegetables, radish, potato etc. are grown in Nepal [11].

### Study population

The eligibility criteria were people aged between 25 and 65 years and listed on the voter list of 2007 in Pokhara Municipality in the Kaski district. People with severe illness such as people whose participation in this study would hamper their health condition and pregnant women were excluded. All participants in the study provided written informed consent before the enrolment.

### Study design and tools

This cross-sectional study conducted in 2015 followed the WHO STEPwise approach to surveillance (STEPS) tool [12]. The tool has been validated and used by Nepal Health Research Council [9]. The tool provides information on physical measurements (height, weight), socio-demographics (age, sex, ethnicity, family size, occupation, income, education, etc.), lifestyle factors (salt consumption, smoking, alcohol, physical activity, etc.), and blood pressure (BP).

### Sample size and sampling procedure

This is a further analysis of COBIN study. The sample size for this study was calculated based on the prevalence of hypertension in Nepal. The description has been discussed in detail elsewhere [10, 13]. In brief, the random sample of participants were selected using voter list of the study municipality. Assuming 25% prevalence of hypertension, 95% confidence intervals (CI) and a 5% margin of error and eight strata based on age and sex group, 2882 participants were approached for data collection. While 2% of the sampled population did not give consent and/or were not available during the household visits, 2815 participants were included in this study, of which 1843 were female and 972 were male who provided complete information on history of self-reported major cardiovascular events and fruit and vegetable intake.

### Variables and definition of variables

#### Socio-demographic characteristics

The socio-demographic variables included in this study were age, ethnicity, sex and educational level. Age was measured in years and classified into four groups (24–34, 35–44, 45–54 and 55–64 years). Ethnicity was sub-grouped into three categories such as upper caste (advantaged ethnic group), janajati (relatively disadvantaged ethnic group) and others in accordance with the coding of the Government of Nepal [14]. Educational level was classified into three categories; primary school, secondary and high school, and college or university.

#### Major cardiovascular events

For self-reported history of myocardial infarction and stroke, respondents were asked a prompt question in Nepali, “Have you ever had a stroke or heart attack?” If the respondent was not sure about their answer, they showed medical records from hospital and/or the bristle pack of medicine used for myocardial infarction and stroke.

#### Fruit and vegetable intake

Data collection on number of servings was done as a part of the WHO STEPS tool. “One cup of raw, leafy green vegetables, half a cup of other cooked or raw vegetables or half a cup of vegetable juice was regarded as one serving of vegetable. One medium-size piece of fruit, half a cup of raw, cooked or canned fruit, or half a cup of juice from a fruit (not artificially flavored) was considered as one serving of fruit [12].” A show card illustrated typical examples of local fruits and vegetables and participants were asked about the number of servings of fruits and vegetables. Each show card illustrated the size of the servings. Based on the show card, participants self-reported their number of daily servings. When the number of fruit and vegetable intake a day was disaggregated into < 5 and ≥ 5 servings, we found that 10.2% of respondents consumed ≥5 servings a day and 89.8% consumed < 5 servings of fruits and vegetables a day. Intake of fruit and vegetable was classified as < 3 and ≥ 3 servings based on the median value (3 servings a day) or mean values of 3.3 servings a day. A former prospective cohort study conducted across 18 countries have also classified fruit and vegetable serving into 3–4 servings and reported that even moderate consumption of fruits and vegetables (equivalent to 3–4 servings a day) resulted in significant reduction of all-cause mortality and lower incidence of cardiovascular diseases [15].

#### Major risk factors of cardiovascular disease

Body mass index (BMI) was calculated by dividing weight (in kg) by square of height (in meters), classified as underweight (< 18.5 kg/m^2^), normal (18.5–24.9 kg/m^2^) and overweight/obese (≥25 kg/m^2^) [16]. In terms of physical activity, < 600 Metabolic Equivalent of Task (MET) minutes/week was considered low, 600–3000 MET minutes/week was considered medium and > 3000 MET minutes/week was considered high [12]. Harmful intake of alcohol was classified yes if men were drinking 15 or more standard drinks a week and if women were drinking 8 or more standard drinks a week [17]. If the systolic BP was 140 mmHg or higher, if the diastolic BP was 90 mmHg or higher or if using antihypertensive medication, then the respondent was considered to have hypertension [18]. Diabetes Mellitus was categorized as “yes” or “no”, based on information provided by health professionals to establish if respondents had been diagnosed with diabetes mellitus within the last 12 months. Respondents were considered current smokers if reporting to currently smoking cigarettes.

### Statistical analysis

Data analysis was performed using the statistical software R version 3.6.3 [19]. Descriptive statistics were carried out to identify the frequency and percentage of respondents according to fruit and vegetable intake. The Chi-square test was used to examine the association between fruit and vegetable intake and history of self-reported cardiovascular events, socio-demographic and cardiovascular risk factors. A *p*-value below 0.05 was considered statistically significant.

Binary logistic regression analysis was used to compute the odds ratio with 95% CI to find out the factors affecting fruit and vegetable intake where fruit and vegetable intake was regarded as outcome variable and socio-demographic (age, sex, ethnicity, education), cardiovascular risk (diabetes mellitus, currently smoking, hypertension, BMI, harmful alcohol consumption, physical activity) factors and history of self-reported cardiovascular events were independent variables. Both crude odds ratio (COR) and adjusted odds ratio (AOR) were reported. No variables were controlled in the crude model and all the independent variables such as socio-demographic (age, sex, education, ethnicity), cardiovascular risk factors (diabetes mellitus, currently smoking, hypertension, BMI, harmful alcohol consumption, physical activity) and history of self-reported cardiovascular events were controlled in the adjusted model (Table 3). Similarly, another binary logistic regression analysis was performed between history of self-reported major cardiovascular events and fruit and vegetable intake to compute the odds of having a history of major cardiovascular events for respondents who consumed < 3 servings of fruits and vegetables in a day compared to those who consumed ≥3 servings of fruits and vegetables in a day. The history of self-reported major cardiovascular events was regarded as outcome variable, fruit and vegetable intake, socio-demographic and cardiovascular risk factors were independent variables. No variables were controlled in the crude model and all the independent variables such as socio-demographic (age, sex, education, ethnicity), cardiovascular risk factors (diabetes mellitus, currently smoking, hypertension, BMI, harmful alcohol consumption, physical activity) and fruit and vegetable intake were controlled in the adjusted model (Table 4).

Data on age, BMI and physical activity were kept as continuous variables in the logistic regression. The likelihood ratio test was performed to compare the two models, i.e. one with the continuous variable and another with the same categorical variable. The *p*-value was more than 0.05 for all the three variables for likelihood ratio test and the null hypothesis was accepted. The model with continuous variable best fits the data and therefore, this model was chosen. No interaction was found between independent variables and fruit and vegetable intake.

### Ethics

The Nepal Health Research council approved the protocol for the original study (reference number 1065). All study participants were informed about study objectives, procedures and their role in the study. Written informed consent was obtained from all participants before data collection.

## Results

Table 1 shows the number of servings of fruits and vegetables a day as well as number of days of eating fruits and vegetables in a typical week. Half (49.3%) of the respondents consumed one serving of fruit on average and 93.3% consumed two servings of vegetables on average per day. The mean and median intake of fruits and vegetables were 3.3 ± 0.79 and 3 servings respectively.

**Table 1:** Fruit and vegetable intake among persons aged 25–65 years respondents from Pokhara Municipality, Nepal, 2015

Fruit and vegetables intake	Frequency %(N)
Number of servings of fruit consumed on average per day (*N* = 2578)	
1	49.3 (1388)
2	41.2 (1159)
3	0.8 (22)
4	0.1 (2)
5	0.0 (0)
≥ 6	0.2 (7)
Number of days fruit was consumed in a typical week (*N* = 2795)	
0	7.6 (214)
1	18.2 (511)
2	22.7 (640)
3	19.9 (561)
4	10.2 (288)
5	5.5 (156)
≥ 6	15.1 (425)
Number of vegetable servings consumed on average per day (*N* = 2809)	
1	5.6 (158)
2	93.3 (2625)
3	0.8 (23)
5	0.0 (1)
≥ 6	0.1 (2)
Number of days vegetables were consumed in a typical week (*N* = 2814)	
0	0.2 (5)
1	0.5 (15)
2	1.6 (44)
3	3.4 (96)
4	7.9 (223)
5	13.0 (365)
≥ 6	73.4 (2066)

Table 2 shows the distribution of respondents according to socio-demographic characteristics, self-reported history of major cardiovascular events and cardiovascular risk factors by number of fruit and vegetable servings a day. Among respondents who consumed < 3 servings of fruits and vegetables a day, 3.4% of respondents reported having a history of major cardiovascular events and 96.6% reported not having a history of major cardiovascular events. Similarly, among those who consumed ≥3 servings of fruits and vegetables a day, 1.9% of respondent reported having a history of major cardiovascular events and 98.1% reported not having a history of major cardiovascular events. Lastly, prevalence of self-reported history of major cardiovascular events was 2%.

**Table 2:** Fruit and vegetable intake by self-reported history of major cardiovascular events, socio-demographic characteristics and cardiovascular risk factors among persons aged 25–65 years respondents from Pokhara Municipality, Nepal, 2015

Characteristics	Number of servings a day % (N)			***p***-value^**$**^
	< 3 servings	≥3 servings	Total	
**Age in years (*N*** **= 2656)**				0.001*
24–34	16.7 (52)	20.4 (479)	531	
35–44	25.4 (79)	30.6 (717)	796	
45–54	29.6 (92)	30.1 (707)	799	
55–64	28.3 (88)	18.8 (442)	530	
**Ethnicity (*N*** **= 2815)**				< 0.001*
Janajati	33.0 (116)	32.8 (808)	924	
Others	30.2 (106)	12.6 (311)	417	
Upper caste	36.8 (129)	54.6 (1345)	1474	
**Sex (*N*** **= 2815)**				0.981
Female	65.5 (230)	65.5 (1613)	1843	
Male	34.5 (121)	34.5 (851)	972	
**Educational level (*N*** **= 2815)**				< 0.001*
Up to primary	68.4 (240)	49.8 (1226)	1466	
Secondary and high school	26.8 (94)	43.7 (1076)	1170	
College or university	4.8 (17)	6.6 (162)	179	
**Harmful alcohol consumption (*N*** **= 2815)**				< 0.001*
Yes	43.9 (154)	34.4 (847)	1001	
No	56.1 (197)	65.6 (1617)	1814	
**Current smoking (*N*** **= 2815)**				< 0.001*
Yes	29.9 (105)	15.1 (372)	477	
No	70.1 (246)	84.9 (2092)	2338	
**History of major cardiovascular events (*N*** **= 2815)**				0.064
Yes	3.4 (12)	1.9 (47)	59	
No	96.6 (339)	98.1 (2417)	2756	
**Hypertension (*N*** **= 2815)**				0.949
Yes	29.9 (105)	29.7 (733)	838	
No	70.1 (246)	70.3 (1731)	1977	
**Diabetes Mellitus (*N*** **= 2815)**				0.839
Yes	4.0 (14)	4.2 (104)	118	
No	96.0 (337)	95.8 (2360)	2697	
**Physical activity (*N*** **= 2815)**				0.453
Low	1.1 (4)	0.6 (14)	18	
Moderate	6.0 (21)	6.1 (150)	171	
High	92.9 (326)	93.3 (2300)	2626	
**BMI (*N*** **= 2815)**				< 0.001*
Underweight	12.5 (44)	5.9 (145)	189	
Normal	52.1 (183)	43.9 (1082)	1265	
Overweight/obese	35.3 (124)	50.2 (1237)	1361	

BMI refers to body mass index. $*p*-value was reported from the Chi-square test performed between fruit and vegetable intake and history of self-reported cardiovascular events, socio-demographic and cardiovascular risk factors. *refers to statistically significant at *p* value < 0.05

Table 3 presents the crude and adjusted odds ratios from the logistic regression analysis for finding out factors affecting the fruit and vegetable intake. Respondents with a secondary-level education had an AOR of 0.55 (95% CI: 0.31–0.97) and those belonging to upper caste ethnicity had an AOR of 0.68 (95% CI: 0.50–0.99) of consuming < 3 servings of fruits and vegetables a day compared to those who consumed ≥3 servings of fruits and vegetables a day. The respondents having a history of cardiovascular events (AOR 2.33; 95%CI: 1.12–4.83) and currently smoking (AOR 1.39; 95%CI: 1.01–1.91) had higher odds of consuming < 3 servings of fruits and vegetables a day compared to those not having history of cardiovascular events and non-smokers respectively. Moreover, with every 1 kg/m^2^ increase in BMI, the odds of consuming < 3 servings of fruit and vegetable was 0.95 (95% CI: 0.92–0.98) times than that of consuming ≥3 servings of fruits and vegetables a day.

**Table 3:** Crude and adjusted odds ratio from logistic regression analysis for identifying factors affecting the fruit and vegetable intake (reference = ≥3 servings) among persons aged 25–65 years respondents from Pokhara Municipality, Nepal, 2015

Characteristics	COR^**a**^ (<3servings)	95% CI		AOR^**b**^ (<3servings)	95% CI	
		Lower Bound	Upper Bound		Lower Bound	Upper Bound
**Age (in years) (*N*** **= 2656)**	1.02	1.01	1.03	1.01	0.99	1.03
**Sex (*N*** **= 2815)**						
Male	1.00	0.79	1.26	0.92	0.66	1.27
Female	Ref					
**Educational level (*N*** **= 2815)**						
Up to primary	1.87	1.11	3.13	0.89	0.48	1.64
Secondary and high school	0.83	0.48	1.43	0.55	0.31	0.97
College/ university	Ref					
**Ethnicity (*N*** **= 2815)**						
Upper caste	0.67	0.51	0.87	0.68	0.50	0.99
Others	2.37	1.76	3.19	1.62	1.15	2.27
Janajati	Ref					
**History of major cardiovascular events (*N*** **= 2815)**						
Yes	1.82	0.96	3.47	2.33	1.12	4.83
No	Ref					
**Diabetes Mellitus (*N*** **= 2815)**						
Yes	0.94	0.53	1.67	0.81	0.41	1.61
No	Ref					
**Hypertension (*N*** **= 2815)**						
Yes	1.01	0.79	1.29	1.00	0.75	1.34
No	Ref					
**Current smoking (*N*** **= 2815)**						
Yes	2.40	1.86	3.09	1.39	1.01	1.91
No	Ref					
**Harmful alcohol consumption (*N*** **= 2815)**						
Yes	1.50	1.19	1.88	1.09	0.80	1.49
No	Ref					
**Physical activity** **(MET/Week)** **(*N*** **= 2815)**	1.00	1.00	1.00	1.00	1.00	1.00
**BMI (Kg/m2)** **(*N*** **= 2815)**	0.91	0.89	0.94	0.95	0.92	0.98

Note: Age, physical activity and BMI are included as continuous variables in the model. COR refers to crude odds ratio, AOR refers to adjusted odds ratio, CI refers to confidence intervals, MET refers to metabolic equivalent of task, BMI refers to body mass index and kg refers to kilogram. Fruit and vegetable intake was the outcome variable and socio-demographic (age, sex, education, ethnicity), cardiovascular risk factors (diabetes mellitus, currently smoking, hypertension, BMI, harmful alcohol consumption, physical activity) and history of self-reported cardiovascular events were independent variables. ^a^No adjustment done in the COR model. ^b^All the independent variables such as socio-demographic (age, sex, education, ethnicity), cardiovascular risk factors (diabetes mellitus, currently smoking, hypertension, BMI, harmful alcohol consumption, physical activity) and history of self-reported cardiovascular events were controlled in the AOR model

Table 4 shows crude and adjusted odds ratios from logistic regression analysis for self-reported history of major cardiovascular events by fruit and vegetable intake, socio-demographic characteristics and risk factors related to cardiovascular diseases. The odds of having a history of major cardiovascular events was 1.82 (95%CI: 0.96–3.47) for respondents who consumed < 3 servings of fruits and vegetables in a day compared to those who consumed ≥3 servings of fruits and vegetables in a day without adjusting for socio-demographic and cardiovascular risk factors. When adjusting for socio-demographic and cardiovascular risk factors, the odds ratio increased to 2.22 (95%CI: 1.06–4.66). A significant association was also noted between hypertension and self-reported history of major cardiovascular events (AOR 9.17; 95%CI: 4.44–18.94).

**Table 4:** Crude and adjusted odds ratio from logistic regression of self-reported history of major cardiovascular events (reference = no cardiovascular events) by fruit and vegetable intake, socio-demographic characteristics and cardiovascular risk factors among persons aged 25–65 years respondents from Pokhara Municipality, Nepal, 2015

Characteristics	COR^**a**^ (Having cardiovascular events)	95% CI		AOR^**b**^ (Having cardiovascular events)	95% CI	
		Lower Bound	Upper Bound		Lower Bound	Upper Bound
**No. of servings a day (*N*** **= 2815)**						
< 3 servings	1.82	0.96	3.47	2.22	1.06	4.66
≥ 3 servings	Ref					
**Age (in years)** **(*N*** **= 2656)**	1.04	1.01	1.07	1.01	0.97	1.04
**Sex (*N*** **= 2815)**						
Male	1.22	0.72	2.07	0.93	0.43	1.98
Female	Ref					
**Educational level (*N*** **= 2815)**						
Up to primary	0.73	0.28	1.90	0.58	0.17	2.05
Secondary and high school	0.73	0.27	1.94	0.75	0.25	2.21
College/ university	Ref					
**Ethnicity (*N*** **= 2815)**						
Upper caste	1.09	0.62	1.93	1.27	0.63	2.55
Others	0.81	0.34	1.95	0.81	0.30	2.21
Janajati	Ref					
**Diabetes Mellitus (*N*** **= 2815)**						
Yes	2.67	1.13	6.35	1.52	0.56	4.09
No	Ref					
**Hypertension (*N*** **= 2815)**						
Yes	8.78	4.72	16.33	9.17	4.44	18.94
No	Ref					
**Current smoking (*N*** **= 2815)**						
Yes	1.26	0.66	2.39	0.10	0.45	2.24
No	Ref					
**Harmful alcohol consumption (*N*** **= 2815)**						
Yes	1.00	0.58	1.72	0.90	0.44	1.85
No	Ref					
**Physical activity** **(MET/Week)** **(*N*** **= 2815)**	1.00	1.00	1.00	1.00	1.00	1.00
**BMI (Kg/m2) (*N*** **= 2815)**	1.01	0.96	1.07	0.97	0.90	1.04

Note: Age, physical activity and BMI are included as continuous variables in the model. COR refers to crude odds ratio, AOR refers to adjusted odds ratio, CI refers to confidence intervals, MET refers to metabolic equivalent of task, BMI refers to body mass index and kg refers to kilogram. History of self-reported cardiovascular events was the outcome variable, fruit and vegetable intake, socio-demographic (age, sex, education, ethnicity) and cardiovascular risk factors (diabetes mellitus, currently smoking, hypertension, BMI, harmful alcohol consumption, physical activity) were independent variable. ^a^No adjustment done in the COR model. ^b^All the independent variables such as socio-demographic (age, sex, education, ethnicity), cardiovascular risk factors (diabetes mellitus, currently smoking, hypertension, BMI, harmful alcohol consumption, physical activity) and fruit and vegetable intake were controlled in the AOR model

## Discussion

The present study suggested an inverse association between intake of fruits and vegetables and self-reported history of myocardial infarction or stroke. The respondents consuming < 3 servings of fruits and vegetables a day had a 2.22 (95%CI: 1.06–4.66) adjusted odds of having a history of myocardial infarction or stroke. Our results support previous studies describing an inverse association between fruit and vegetable consumption and ischemic stroke [20, 21]. Higher intake of fruits and vegetables was associated with lower risk of stroke in both studies [20, 21]. Interestingly, a prospective cohort study conducted across 18 countries reported that even moderate consumption of fruits and vegetables (equivalent to 3–4 servings a day) resulted in significant reduction of all-cause mortality and lower incidence of cardiovascular diseases [15].

The current study also demonstrated a significant association between hypertension and self-reported history of cardiovascular events. This is consistent with the results of a former study [22] showing that hypertension was an important risk factor of cardiovascular diseases.

Despite WHO recommendations of consuming ≥5 servings of fruits and vegetables a day, the mean daily consumption of fruits and vegetables is inadequate especially in low- and middle-income countries like Nepal [23]. WHO has previously reported an average of 3.17 servings (2.99–3.35) per day in low- and middle-income countries [23]. On the other hand, we reported a slightly higher consumption in our study with an average of 3.3 servings per day.

We also found that the intake of fruits and vegetables varied among different socio-demographic variables. Respondents belonging to upper caste ethnicity were less likely to consume < 3 servings of fruits and vegetables a day (AOR 0.68; 95% CI: 0.50–0.99) compared to janajati. Similar to this result, a study conducted in India reported that the overall food consumption pattern was lower among people belonging to the lower caste group [24]. Upper caste people are moderately better educated [25] and wealthier than those belonging to other ethnicities [26]. Educational level was significantly associated with fruit and vegetable intake (*p* < 0.001). Respondents who had a secondary-level education were less likely to consume < 3 servings of fruits and vegetables with an AOR of 0.55 (95% CI: 0.31–0.97) than those who had attended college or university. This corroborates results of a South Asian study reporting that less educated people were less likely to consume adequate amounts of fruits and vegetables [27]. Thus, the policies and interventions targeted to increase consumption of fruits and vegetables should be designed as per the socio-demographic gradient of Nepal. Another study from same study area reported that individual lifestyle counselling alone would not help to consume more fruit and vegetable intake among the participants [28]. Thus, further research encompassing policies, market and economics are also needed to develop further plan and policies, which can be translated in the real scenario.

The prevalence of self-reported history of myocardial infarction and stroke was 2% in this study. This result is comparable to the result of a national survey done in Nepal in 2019 stating that 1.1% of adults aged 15–69 years reported ever having experienced a heart attack, angina or stroke [29]. Nepal has committed to a 25% relative reduction in premature death from non-communicable diseases by 2025 [30]. However, Nepal is far behind translating guidelines to practice, specifically focusing dietary behaviors and practices. The National Nutrition Policy Strategy of 2004 recommends consuming a variety of fruits and vegetables [31]. The Multi-sectoral Action Plan for the Prevention and Control of Non-communicable diseases (2014–2020) encourages consumption of fruits and vegetables and discourages consumption of unhealthy foods [30] to prevent cardiovascular diseases; however, no specific guidelines are available.

### Strengths and limitations

This is the first study from Nepal reporting the association between current fruit and vegetable intake and self-reported history of major cardiovascular events using population-based random samples. However, limitations should be considered while interpreting results. Firstly, a causal relationship could not be established due to the cross-sectional nature of the study. Further prospective studies are needed to explore the causal pathway between fruit and vegetable intake and major cardiovascular events in the Nepali population. Another limitation of this study is that both exposure and outcome variables were self-reported [7]. The information on duration of MI/stroke in the study is not known. Thus, we cannot establish that if the current trend on consumption of fruits and vegetables is due to recent cardiovascular events or culmination of behavior due to past cardiovascular events. The information on fruits and vegetables intake was based on self-reported 24-h and 1 week recalls, which may result in some recall bias; however, recall bias would have been minimized by using show cards and local languages for the type of fruits and vegetables and their units. Further, it was not possible to exclude potatoes and other tuberous roots from the total vegetable intake because both of them are strongly considered as vegetables in Nepal. Another important limitation of the study is that it did not take seasonal variation in the account. The availability and consumption of different types of fruits and vegetables in Nepal varies according to the season [11], which may underestimate or overestimate the intake of fruit and vegetable according to the season the data was collected.

## Conclusion

The study reported that respondents currently smoking, with low BMI, having a history of cardiovascular events and belonging to other ethnicity except upper caste and janajati are significantly associated with lower consumption of fruit and vegetable intake. It was found that participants who consumed < 3 servings of fruits and vegetables a day had higher odds of having a history of myocardial infarction or stroke in comparison to those who consumed ≥3 servings of fruits and vegetables a day. This finding may be useful as a policy recommendation for those settings, where the current WHO recommendation of ≥5 servings of fruits and vegetables is not possible. This association suggests that even patients with prior myocardial infarction or stroke do not modify nutritional intake according to guidelines. Additionally, the overall low intake of fruits and vegetables in Nepal is a risk factor for cardiovascular disease progression in Nepal.

## Data Availability

The dataset used in this study is available upon reasonable request to COBIN study group principle investigator Dinesh Neupane (last author), who can be contacted through neupane.dinesh@gmail.com.

## Abbreviations

AOR: Adjusted odds ratio
BMI: Body mass index
BP: Blood pressure
CI: Confidence intervals
COBIN: Community based Management of non-communicable diseases in Nepal
COR: Crude odds ratio
MET: Metabolic equivalent of task
WHO: World Health Organization
STEPS: STEPwise approach to surveillance tool
